# Tackling Health Inequalities in Cancer Screening: Experiences of Eastern European and African Communities in Northeast Scotland

**DOI:** 10.1002/cam4.71236

**Published:** 2025-09-15

**Authors:** Sarah R. Prowse, Shaun Treweek, María José Pavez Larrea, Miriam Brazzelli, Adriana Uribe

**Affiliations:** ^1^ Aberdeen Centre for Evaluation University of Aberdeen Aberdeen UK; ^2^ Grampian Regional Equality Council Ltd Aberdeen UK

**Keywords:** cancer screening, delivery of health care, early detection of cancer, health services research, medically under‐served populations

## Abstract

**Introduction:**

This report provides insights from ethnic minority community members who may be under‐served by the healthcare system in Northeast Scotland, focusing on how to improve the delivery of cancer screening programmes.

**Methods:**

A partnership approach was used to engage the public with particular emphasis on reaching Polish/Eastern European and African communities.

**Results:**

Key areas for improvement identified through discussion groups include communication, cultural competence, access to screening services, and knowledge‐raising initiatives to help individuals navigate the UK healthcare system.

**Conclusions:**

These findings offer recommendations as to how healthcare systems can engage with diverse communities for meaningful reform within cancer screening programmes.

## 1 Introduction

In the United Kingdom (UK), the National Health Service (NHS) provides population‐based screening programmes for bowel, breast, and cervical cancer that are free to access and aim to detect cancer or precancerous activity in asymptomatic individuals when it is at an earlier and more treatable stage of disease [1]. Within NHS Scotland (Grampian), which serves northeast Scotland, a need has been identified to better engage with under‐served groups most at risk of not participating in preventative cancer screenings. This includes Polish/Eastern European groups, as well as the African community broadly, with a focus on communities living in rural or remote areas of the Grampian region [2].

Patient and public involvement (PPI) has been found to generate high‐quality, relevant insights that effectively address the needs of diverse populations when considering the uptake of cancer screening services [3]. However, for PPI to be truly meaningful in communities under‐served by current research, engagement strategies must foster genuine partnerships that enable diverse groups to have a real influence on health services [4]. To support this approach, we collaborated with the Grampian Regional Equality Council (GREC), a community partner with extensive experience in PPI initiatives relevant to under‐served communities within the Grampian health region [5].

This report includes a brief summary of discussions with predominantly Polish/Eastern European and African communities in Aberdeen, Aberdeenshire, and Moray, Scotland on how the NHS could improve the delivery of cancer screening programmes. This work adheres to the National Institute for Health and Care Research definition of PPI, where community members act as specialist advisors to provide valuable knowledge and expertise based on their experience of a public health concern [6]. These shared insights provide a further basis for how healthcare systems, both in the UK and globally, can engage with diverse communities for meaningful reform within cancer screening programmes to better reach those who are currently under‐served.

## 2 Methods

The PPI initiative was informed by a previously commissioned overview of international research published in 2024 highlighting the barriers and facilitators to the uptake of cancer screening services [1].

Six discussion groups were recruited and facilitated by GREC Community Connectors, who are staff with in‐depth, lived experience of the target communities and direct links to ethnic minority groups. This enabled a culturally sensitive and trust‐based approach, with those invited purposefully engaged from within the target communities, rather than being selected from a general pool of volunteers. No formal inclusion or exclusion criteria were applied to discussion groups beyond general eligibility for NHS Scotland cancer screening invitations. However, invitations were extended by Community Connectors to reflect a diversity of experiences within each community, including variation in age, gender and prior engagement with screening. As this was an early‐stage PPI activity, variables including language or length of residency were not used to stratify invitations, but rather the community‐led nature of the process allowed for the inclusion of community members with a broad range of relevant perspectives and experiences.

Four discussion groups were conducted in‐person in Aberdeen, while two were held online with a focus on the more rural communities of Aberdeenshire and Moray. Five of the six discussion groups were successfully recorded. The sixth group, conducted online, faced technical problems, preventing the recording from being arranged. Translation and interpretation were available via the relevant facilitators. Participation in the discussion groups was voluntary. Consent was not required as the discussion groups informed and advised on future research with individuals acting as specialist advisers, not research participants, and provided valuable insights based on their experiences with cancer screening services. Discussions were audio‐recorded, and comments aligned with the themes identified in the earlier published overview of international research [1].

## 3 Results

Each discussion group lasted approximately 1–1.5 h in length. A total of 30 participants took part in the discussion groups, with the majority (25/30, 83%) identifying as women. This gender difference reflects the screening programmes discussed, as two of the three (breast and cervical) are largely targeted at those the NHS identifies as women and/or people with a cervix.

The demographic composition of the groups reflected our efforts to engage with a wide range of community members to ensure inclusivity and diversity in our discussions. Those involved came from diverse cultural backgrounds, with the majority from the Polish/Eastern European (13/30, 43%) and African (11/30, 37%) communities.

The key factors influencing the uptake of cancer screening identified by the discussion groups included: aspects of communication, attitudes towards screening, differing experiences and preferences between screening globally and the UK, resources including issues of appointment attendance, and factors related to the social acceptance of screening.

Further reflections within the discussion groups yielded several insights as presented in Table 1. We implemented a RAG (Red‐Amber‐Green) rating system to assess each insight, where red indicates complex, resource‐intensive solutions, amber reflects moderate feasibility, and green represents achievable solutions that may be practical to implement in the short term with immediate impact. These proposed areas for improvement apply to both the NHS and other global health systems that offer routine cancer screening services.

**TABLE 1:** RAG ratings of key insights from the discussion groups to increase the uptake of cancer screening services.

Key insight(s)	Summary of responses	Considerations for the healthcare system	RAG rating	Rationale
Cancer screening services are not easily accessible	Improvements are needed in the practicalities of accessing screening services, notably for rural and/or remote communities	Suggestions included flexible online appointment booking systems and convenient screening locations, such as pop‐up clinics; the role of transportation should also be considered	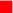 Red	Requires high effort changes to infrastructure and the design and delivery of services, including logistics, resource allocation, and system coordination
Processes within the healthcare system lack clarity and are not always easily understood	Newcomers may still prefer to access health systems within their home country as the NHS lacks support for those needing to navigate a foreign healthcare system, including accessing cancer screening services and understanding wider healthcare pathways	Supporting engagement efforts may include written materials specific to the health region with applicable translation and workshops/community events emphasising health advocacy and building trust with the health system	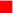 Red	Involves substantial changes to current systems, processes and organisational structures
Diverse groups have unmet needs within cancer screening services and the wider healthcare system (no ‘one‐size‐fits‐all’ approach)	Emphasis on the importance of culturally sensitive approaches to cancer screening including consideration of beliefs, practices, and language within diverse communities	Overlap with the need for strong interpersonal skills of healthcare professionals and practitioners that reflect the lived experiences of patients (including gender, notably as relevant to aspects of faith)	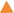 Amber	Potential for high impact change as ongoing structural and workforce developments continue to address these challenges and improve service delivery
Better awareness and knowledge initiatives surrounding cancer generally are needed	Identified need for more information regarding cancer risk factors, prevention strategies, and screening options (including self‐testing)	Initiatives such as community workshops and peer‐to‐peer support networks could provide educational content and resources to empower individuals with knowledge about cancer and the importance of screening and early detection	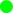 Green	Feasible with ongoing community outreach; proven high value solutions when local communities are actively and positively engaged with the healthcare system
Invitations to attend cancer screening(s) can be improved	Feedback overwhelmingly indicated that the content of invitation letters for cancer screening programmes could be improved to better communicate the importance and benefits of participation	Use positive messaging (screening as a means of good health) versus avoiding negative consequences (including death); translated materials should be available whenever possible, and use of pictures is preferred	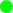 Green	Clear, actionable suggestions that could be trialled or implemented with low effort for potentially high impact

*Note:* A square for red, a triangle for amber, and a circle for green.

## 4 Discussion

Overwhelmingly, the need for better communication regarding screening services was the key highlight in feedback provided by both African and Polish/Eastern European community members. Invitation letters sent via the postal system are one of the most frequently used global evidence‐based approaches to promoting cancer screening participation [7]. The choice of wording used in the invitations was of concern for members of the African community, who indicated that they would be more receptive to positive messaging focusing on the benefits of screening rather than information perceived as negative and/or scary, including mentions of overall mortality from cancer(s).

Language was a crucial factor for many, starting with the initial invitation through to the importance of translation services available via the healthcare system. On‐site translation services were reported as an essential factor in the uptake of cancer screening services. All discussion groups emphasised the importance of culturally sensitive approaches to cancer screening, including knowledge of the beliefs, practices, and languages within diverse communities.

Community members often preferred care providers who shared their lived experiences. This included providers who spoke the same language, shared a cultural background including faith, and, in the case of breast and cervical screening programmes, a preference for female providers. A recent co‐designed faith‐based intervention for Muslim women in Scotland aiming to increase the uptake of all three cancer screening programmes highlighted that cultural barriers or a lack of awareness often impeded participation more than religious barriers alone [8]. This finding was echoed by some discussion group members, who valued the role of faith‐based and community leaders in promoting awareness of cancer and cancer screening services.

Members from both the African and Polish/Eastern European communities expressed a desire for increased education and awareness initiatives to promote the importance of early detection and regular screening for cancer. Members of the African community in particular noted the importance of social support to encourage attendance. This highlights an area where initiatives such as community‐led workshops could provide educational content and resources to empower individuals with knowledge about cancer and its potential consequences. Local partnerships, such as those with GREC, are essential for implementing and driving long‐term, fit‐for‐purpose change within broader health systems and highlight the importance of PPI initiatives at the outset of the planning process.

Responses from Polish/Eastern European members reflected a need for clearer insights into navigating a foreign healthcare system. Some individuals admitted returning to Poland or other countries of origin to attend services such as cancer screenings. The quality of care received in these countries was often viewed as superior to that offered in the UK. The Cancer Strategy for Scotland 2023–2033 sets out a 10‐year vision to improve national cancer survival and care and addresses many of the key concerns highlighted by the discussion groups such as earlier and faster diagnosis, person‐centred care for all, and a sustainable and skilled workforce [9].

The recommendations from this PPI initiative are relevant not only to the UK but also to global health services similar to the UK NHS. Broader insights, such as the importance of trust in healthcare providers, language accessibility, cultural alignment and structural barriers to cancer screening participation, are relevant across a wide range of health systems. While operational mechanisms may differ, these underlying concepts have global applicability and may inform locally appropriate adaptations, including in contexts where cancer screening is more opportunistic or hospital‐based.

Active engagement with local communities and the public can help address unmet needs in preventing cancer mortality by providing actionable, empowered, and culturally competent health insights. A better understanding of the experiences and needs of under‐served groups enables policymakers, healthcare professionals, and service providers to respond with tailored strategies for addressing inequalities [10].

## Author Contributions

**Sarah R. Prowse:** conceptualization, writing – original draft, methodology, project administration, formal analysis, data curation, validation. **Shaun Treweek:** conceptualization, funding acquisition, project administration, methodology, writing – review and editing, supervision, resources, formal analysis, validation. **María José Pavez Larrea:** conceptualization, writing – review and editing, methodology, project administration, investigation. **Miriam Brazzelli:** conceptualization, writing – review and editing, methodology. **Adriana Uribe:** conceptualization, investigation, methodology, project administration.

## Ethics Statement

This work adheres to the National Institute for Health and Care Research (NIHR) guidance on patient and public involvement in health and social care research in which active involvement of members of the pubic does not raise any ethical concerns. Members of the public acted as specialist advisors and provided valuable knowledge and expertise based on their experience of a public health concern. Therefore, ethical approval was not required for the active involvement element of the engagement initiative as those involved were not acting as research participants.

## Conflicts of Interest

The authors declare no conflicts of interest.

## Data Availability

The data generated during this work are not publicly available due to privacy and confidentiality restrictions. Please contact the corresponding author for more information.
